# Characterization of copulatory courtship song in the Old World sand fly species *Phlebotomus argentipes*

**DOI:** 10.1038/s41598-020-61867-6

**Published:** 2020-03-20

**Authors:** Alejandra S. Araki, Reginaldo P. Brazil, James G. C. Hamilton, Felipe M. Vigoder

**Affiliations:** 1 Laboratório de Biologia Molecular de Insetos, Instituto Oswaldo Cruz, FIOCRUZ, Rio de Janeiro, 21040-900 RJ Brasil; 2 Laboratório de Doenças Parasitárias, Instituto Oswaldo Cruz, FIOCRUZ, Rio de Janeiro, 21040-900 RJ Brasil; 3 Division of Biomedical and Life Sciences, Faculty of Health and Medicine, Lancaster University, Lancashire, LA1 4YG United Kingdom; 4 Universidade Federal do Rio de Janeiro, Instituto de Biologia. Avenida Carlos Chagas Filho, 373, Block A/A2-075.Cidade Universitária/CCS - Centro de Ciências da Saúde, 21941902 Rio de Janeiro, Brazil

**Keywords:** Behavioural ecology, Animal behaviour

## Abstract

Acoustic communication in the form of courtship and mating songs are often involved in reproductive isolation between species of Diptera, such as Drosophila, mosquitoes and sand flies. The patterns of courtship songs in New World sand fly species evolve quickly under sexual selection; and therefore, represent an important trait that can be used as a marker to study the evolution of species complexes and may aid identification of sibling species with a complex. The ability to identify vector species within species complexes is of critical importance for effective and efficient vector control programs. Species-specific song patterns seems to contribute to reproductive isolation in New World sand fly species, suggesting that auditory communication signals may be widespread among these important vectors of leishmaniasis. The main goal of the present study was to characterize the copulatory courtship song of *Phlebotomus argentipes*, an important vector of visceral leishmaniasis in the Old World. *Ph. argentipes* males produce acoustic signals during copulation and two types of songs were observed. The one we called primary song is a ‘pulse song’ with similar length and amplitude to the previously observed ‘P1’ pattern recorded in Brazilian populations of *Lu. longipalpis s.l*. The secondary song has ‘sine song’ characteristics and is quite different from any song produced by New World species. The discovery of this copulation courtship songs in *Ph. argentipes* supports the possibility that acoustic communication in sandflies might be more widespread than previously thought, including Old World species. Our results highlight the importance of further research on acoustic communication in the *Ph. argentipes* species complex and other Old World vectors of leishmaniasis.

## Introduction

Acoustic signaling represents one of several methods of insect communication and can be used as a defense mechanism in male-male competition and for male-female intra-specific recognition^1,2^. When associated with mating behavior, songs are frequently under sexual selection and thus can diverge quickly^3–5^. In *Drosophila* species, differences in acoustic signals are often associated with pre-mating reproductive isolation and represent sexual traits that result in restricted gene flow between closely related species^6^. Moreover, acoustic communication studies have played a key role in the identification of cryptic sibling species, and therefore, can provide species-specific traits for taxonomic studies when song are associated with reproductive success^7,8^.

*Lutzomyia longipalpis s.l*. Lutz & Neiva 1912 is known to consist of a number of cryptic species that are morphologically indistinguishable from each other^9^. Males of this species produce acoustic signals by flapping their wings. Usually acoustic signals associated with reproductive behavior are produced during pre-mating courtship, as in most *Drosophila* species, and these signals are important for reproductive success^1^. Unlike *Drosophila*, males of *Lu. longipalpis s.l* produce songs after copulation has started, e.g. once the male genital clasps the female genitalia^8,10–12^. Although not very common, copulatory courtship has been reported in some insect groups^13–17^. In the case of *Lu. longipalpis s.l*, copulatory courtship songs are likely to be involved in insemination success as many mating experiments have demonstrated high levels of insemination failure following copulation between sibling species that produce different songs^8,10–12^.

Two types of song have been observed in the *Lu. longipalpis* species complex: a “primary” song which is a pulse song produced by all males during copulation and a “secondary” song composed of low amplitude polycyclic pulses with variable intervals that are produced by some (but not all) males. The primary copulatory courtship song varies considerably among Brazilian populations of *Lu. longipalpis s.l* with three patterns already identified: Burst-type, Pulse-type and Mix-type^18^. The song variation observed suggests that the *Lu. longipalpis* species complex in Brazil consists of at least six cryptic species^19^.

Males from other New World sandfly species also produce acoustic signals. The main vector of visceral leishmaniasis (VL) in the Central-West region of Brazil is *Lu. cruzi* Mangabeira 1938. In this species, which belongs to the *Lu. longipalpis* species complex^20^, males produce a Burst-type copulation song similar to the Burst-type songs produced by some of the *Lu. longipalpis s.l*. sibling species^19^.

Copulation songs have also been found in sandfly vectors of American cutaneous leishmaniasis (ACL). Males of *Lutzomyia migonei* França 1920 produce short ‘trains’ which consist of a small number of pulses with each train exhibiting a short inter-pulse interval. This pattern is different from the Pulse songs observed in the *Lu. longipalpis* species complex^21^. Moreover, pulse songs consisting of short trains were also recorded in males of *Lu*. (*Nyssomyia*) *intermedia* Lutz & Neiva 1912, one of the main vectors of ACL. Unlike the species mentioned previously, *Lu. intermedia* males produce pre-copulatory courtship acoustic signals similar to those observed in several species of Drosophila^22,23^. These observations suggest that acoustic communication is widespread in New World sand flies.

In South East Asia, *Phlebotomus* (*Euphlebotomus*) *argentipes* (Diptera: Culicidae) Annandale & Brunette 1908, is the vector of the Protist *Leishmania donovani* (Kinetoplastida: Trypanosomatidae)^24^. It is thought that member from the *Phlebotomus* and *Lutzomyia* genera have separated approximately 200 MYA^25^. Both the Old World *Ph. argentipes* and the New World *Lu. longipalpis* species display lekking and pheromone communication behavior as part of their mating behavioral repertoire and males have been observed in the field displaying wing fanning^26–29^. Our aim, therefore, was to determine if acoustic signaling may also take place in the Old World leishmaniasis vector, *Ph. argentipes*.

## Results and Discussion

It is known that *Ph. argentipes* males, like *Lu. longipalpis s.l*. males, do not produce a courtship song similar to those that have been observed in *Drosophila* species^22,30^. *Lutzomyia intermedia* is the only sand fly species that is known to produce a song during courtship.

**Figure 1:**
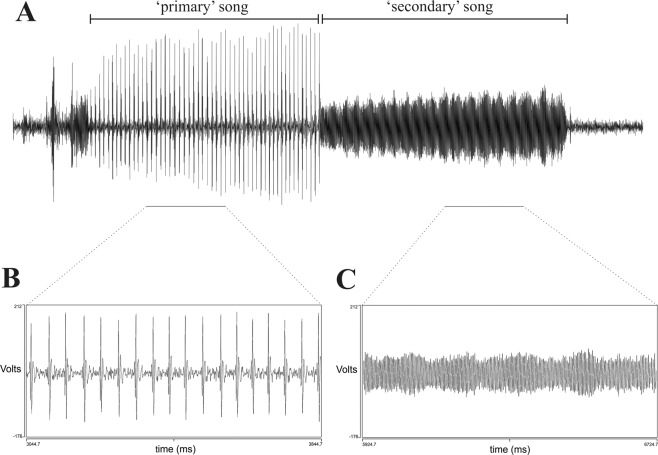
Copulatory courtship song of *Ph. argentipes*. (**A**) General view of song trace showing primary followed by a secondary song trace, (**B**) Trace of a pulse-type primary song and (**C**) Trace of a sine song of the secondary song. Figures (**B**,**C**) show 1 second of song.

**Figure 2:**
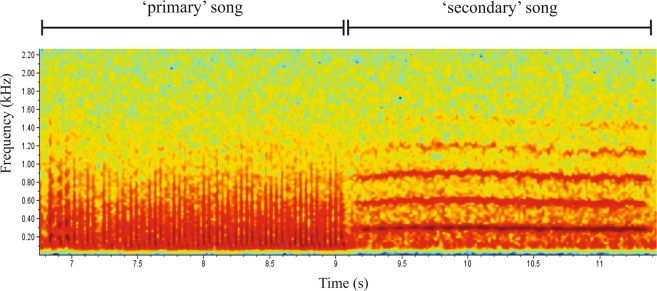
Song spectrogram of copulatory courtship song of *Ph. argentipes*. Pulses with similar length and amplitude compose the primary song and multiple harmonics with five different frequencies are evident in the secondary song.

The study presented here shows that *Ph. argentipes* males produce acoustic signals during copulation, which have similarities to the *Lu. longipalpis s.l*., *Lu. migonei* and *Lu. cruzi* copulation songs^19–21,31^. Only males were observed to produce songs. They produced two types of song: a primary song which is a pulse song, and a secondary song which has sine song characteristics (Fig. 1). Pulse songs consist of trains of uni- or polycyclic sound pulses and sine songs are continuous, humming-like sounds^32^. Figure 2 shows the song spectrograms of both types of songs (audio file:Additional File 1).

The primary song is composed of pulses with similar length and amplitude and shares the qualitative properties of the Pulse-type song observed in some Brazilian populations of *Lu. longipalpis s.l*, e.g. the P1 pattern recorded previously in populations from Jacobina, Jequié and Cavunge (Bahia State). Each train is composed of 43 to 83 pulses (mean 58 ± 3.6), with a mean inter-pulse interval (IPI) of 54.9 ms (SEM ± 4.21), a length of 3.1 s (SEM ± 0.29) and a mean of frequency of 246.8 Hz (SEM ± 15.44) (Additional File 2; Fig. 1B).

The secondary song follows immediately after the primary song and has a mean frequency of 313.1 Hz (SEM ± 7.36) and lasts approximately 2.3 s (SEM ± 0.21) presenting multiple harmonics that resemble the flight sound observed in several mosquito species^33,34^ (Additional File 2; Fig. 1C; Additional File 3). The *Ph. argentipes* secondary song presents more differences when compared to the New World species counterpart. Some male *Lu. longipalpis s.l*. also produce a secondary song^18,31^, however, the pattern is quite different to the one observed in *Ph. argentipes*. The *Lu. longipalpis s.l*. secondary song is more of a pulse-like song with polycyclic pulses that are flanked by two primary songs, and it is not produced by every male. On the other hand, the secondary song of *Ph. argentipes* was produced by every male that we examined (n = 13). Both the primary song and secondary song are produced only once in each copulation interaction, unlike *Lu. longipalpis s.l*., where males can produce each song multiple times during the same copulation sequence^31^.

Sexual signaling controls the exchange of sensory information between partners and plays a direct role in divergence and speciation^22,35^. For example, in the *Drosophila montium* species subgroup the sine song frequency was suggested to be a cue indicating differences among sibling species^36^. In the *Lu. longipalpis* species complex the same function may be performed by the pulse and burst pattern songs^18,19,31^. *Phlebotomus argentipes s.s*. belongs to a species complex and although there are two nominotypical members, *Ph. annandalei* Sinton 1923 and *Ph. glaucus* Mitra & Roy 1953, which can be distinguished by morphological characters^37^, the full extent of the species complex is unclear. It would be interesting to analyze the songs of males from populations of the known species complex members as well as within *Ph. argentipes s.s*., particularly in areas of VL transmission, to evaluate the possibilities that acoustic signals are involved in reproductive isolation within this species.

The study of acoustic communication in vector insects, such as mosquitoes and sandflies, can provide a useful tool in vector control programs, such as the potential to design sound traps or for the assessment of male mating competitiveness in relation to control based on modified male release programs in the field.

## Conclusions

Our results show that *Ph. argentipes* males produce copulatory courtship songs. Two types of patterns are observed, a primary song similar to P1 subtype previously described in a sibling species of the *Lu. longipalpis* species complex, and a subsequent secondary sine song that has not been seen previously in *Lu. longipalpis*. Our analysis represents the first report of the acoustic signals produced during copulation in *Ph. argentipes* and supports the idea that acoustic communication might be widespread in sandflies, including the Old World species. Future study is required to identify song patterns in other putative members of the *Ph. argentipe*s species complex and to determine whether copulatory courtship song is important for sexual communication in Old World sandflies.

## Methods

The *Ph. argentipes* specimens used in this study were obtained from a colony maintained at Keele University, UK, for more than 40 generations at 27 °C, 95% RH, under a 12:12 light:dark photocycle. The colony originated from wild-stock collected near Pune, India, on the east side of India, in a region where there is no visceral leishmaniasis, also known as kala-azar. Recordings were performed according to Souza *et al*.^11^. A virgin male and a virgin female between 2–7 days old were placed inside a small square acrylic chamber (1 cm × 1,5 cm × 0,5 cm) for up to 5 min at 25 ± 1 °C. Recordings were made during afternoon hours until dusk using an INSECTABOX^38^ and a Sony Hi8CCD-TRV65 video camera and Panasonic DMRES10 DVD recorder. Songs were analyzed using DataView version 10.3.0 (Heitler W.J. University of St Andrews, Scotland). Four parameters were measured: (1) IPI, inter-pulse interval; (2) NP, pulses per train; (3) TL, train length; and (4) Freq, the carrier frequency of the pulse train. An Additional file shows the parameters per each individual used for the mean value estimates [see Additional File 2]. The song spectrogram was obtained from Raven version 2.0^39^.

## Supplementary information

Supplementary information

Supplementary information2
