# Supplementary material for: Characterization of copulatory courtship song in the Old World sand fly species Phlebotomus argentipes
Source: Sci Rep. 2020 Mar 20;10:5116. doi: 10.1038/s41598-020-61867-6 (PMC7083918; doi:10.1038/s41598-020-61867-6)

**Supplementary information**

Manuscript: “Characterization of copulatory courtship song in the Old World sand fly species *Phlebotomus argentipes”*

Alejandra S. Araki, Reginaldo P. Brazil, James G.C. Hamilton, Felipe M. Vigoder

**Additional file 2**

Primary and secondary song parameters of *Ph. argentipes*

| | | Primary song | | | | Secondary song | |
|---|---|---|---|---|---|---|---|
| Individual | N | IPI (ms) | NP | TL (s) | Freq (Hz) | Freq (Hz) | TL (s) |
| 119_06 | 1 | 48.83 | 49 | 2.33 | 258.39 | 279.932 | 3079.4 |
| 120_01 | 1 | 58.51 | 50 | 2.87 | 333.77 | 258.398 | 2264.9 |
| 120_03 | 1 | 47.15 | 43 | 1.97 | 236.87 | 312.231 | 2183.5 |
| 120_06 | 1 | 64.57 | 62 | 3.92 | 183.03 | 301.465 | 3277.0 |
| 120_18 | 1 | 97.10 | 57 | 5.41 | 215.33 | 290.698 | 3051.6 |
| 120_19 | 1 | 58.62 | 82 | 4.73 | 290.69 | 333.765 | 3151.6 |
| 120_21 | 1 | 46.52 | 59 | 2.68 | 312.23 | 322.998 | 1443.1 |
| 121_02 | 1 | 44.79 | 83 | 3.66 | 247.63 | 301.465 | 1354.6 |
| 121_03 | 1 | 47.29 | 53 | 2.44 | 193.79 | 322.998 | 2668.0 |
| 121_06 | 1 | 43.22 | 56 | 2.37 | 172.27 | 344.531 | 2715.6 |
| 121_10 | 1 | 48.12 | 69 | 3.26 | 226.09 | 344.531 | 2583.7 |
| 121_11 | 1 | 67.75 | 47 | 3.10 | 204.57 | 344.531 | 873.3 |
| 121_12 | 1 | 41.27 | 44 | 1.76 | 333.77 | 313.129 | 1750.4 |

N, number of train per copula; IPI, inter-pulse interval; NP, pulses per train; TL, train length; Freq, the carrier of frequency of the pulse train.

**Additional file 3**

Detail of trace of a sine song of the secondary song.

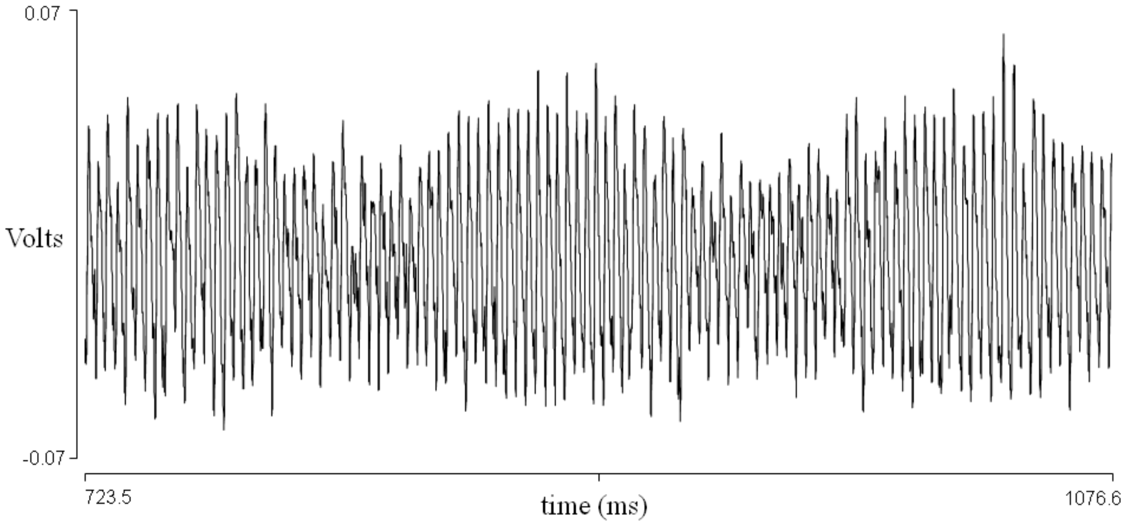

Supplement: Supplementary file 2 — Supplementary information2 [file 41598_2020_61867_MOESM2_ESM.pdf]
